# Transplanted miR-219-overexpressing oligodendrocyte precursor cells promoted remyelination and improved functional recovery in a chronic demyelinated model

**DOI:** 10.1038/srep41407

**Published:** 2017-02-01

**Authors:** Hong-Bin Fan, Li-Xia Chen, Xue-Bin Qu, Chuan-Lu Ren, Xiu-Xiang Wu, Fu-Xing Dong, Bao-Le Zhang, Dian-Shuai Gao, Rui-Qin Yao

**Affiliations:** 1 Department of Cell Biology and Neurobiology, Xuzhou Key Laboratory of Neurobiology, Jiangsu Key Laboratory of New Drug Research and Clinical Pharmacy, Xuzhou Medical University, Xuzhou, 221009, China; 2 Department of Neurology, Affiliated Hospital of Xuzhou Medical University, Xuzhou, 221002, China; 3 Clinical Laboratory, Xuzhou Center Hospital, Xuzhou, 221000, China; 4 Department of Laboratory, No. 100 Hospital of CPLA, Suzhou, 215007, China

## Abstract

Oligodendrocyte precursor cells (OPCs) have the ability to repair demyelinated lesions by maturing into myelin-producing oligodendrocytes. Recent evidence suggests that miR-219 helps regulate the differentiation of OPCs into oligodendrocytes. We performed oligodendrocyte differentiation studies using miR-219-overexpressing mouse embryonic stem cells (miR219-mESCs). The self-renewal and multiple differentiation properties of miR219-mESCs were analyzed by the expression of the stage-specific cell markers Nanog, Oct4, nestin, musashi1, GFAP, Tuj1 and O4. MiR-219 accelerated the differentiation of mESC-derived neural precursor cells (NPCs) into OPCs. We further transplanted OPCs derived from miR219-mESCs (miR219-OPCs) into cuprizone-induced chronically demyelinated mice to observe remyelination, which resulted in well-contained oligodendrocyte grafts that migrated along the corpus callosum and matured to express myelin basic protein (MBP). Ultrastructural studies further confirmed the presence of new myelin sheaths. Improved cognitive function in these mice was confirmed by behavioral tests. Importantly, the transplanted miR219-OPCs induced the proliferation of endogenous NPCs. In conclusion, these data demonstrate that miR-219 rapidly transforms mESCs into oligodendrocyte lineage cells and that the transplantation of miR219-OPCs not only promotes remyelination and improves cognitive function but also enhances the proliferation of host endogenous NPCs following chronic demyelination. These results support the potential of a therapeutic role for miR-219 in demyelinating diseases.

Progressive myelin loss within the central nervous system (CNS), known as demyelination, occurs as a consequence of oligodendrocyte death in diseases such as multiple sclerosis (MS)^1^. Although considerable remyelination is achieved by endogenous progenitor cells, the extent and quality of remyelination is limited. These limitations may arise because oligodendrocyte precursor cells (OPCs) fail to repopulate areas of demyelination or because they are unable to generate remyelinating oligodendrocytes due to the presence of inhibitory factors and/or a lack of the stimuli required to activate these cells to generate remyelinating oligodendrocytes^2^,^3^. Recently, studies involving the transplantation of different cell types (such as neural stem cells, Schwann cells, olfactory ensheathing cells, mesenchymal stem cells, and OPCs) into animal models of demyelination have shown promising results in enhancing myelin repair through multiple mechanisms, including cell replacement, trophic support, immunomodulation, and remyelination^4^,^5^,^6^,^7^,^8^. These studies have suggested that remyelination is a key mechanism in promoting functional recovery following demyelination.

OPCs are found in the adult human brain, constitute 5–8% of total glial cells, and are effective in experimental models of both congenitally dysmyelinated and adult demyelinated brains after transplantation^9^,^10^. The transplantation of ESC-derived OPCs has been shown to promote myelination and neurological function in some CNS disease or injury models such as those of spinal cord injury^11^,^12^, but some cases demonstrate no significant improvement because of limited cell survival, differentiation and migratory ability in an adverse mciroenviroment^13^. Thus, ESCs may serve as an unlimited experimental and therapeutic source of transplantable cells. Although different protocols for the differentiation of OPCs from ESCs have been reported, the efficiency of OPCs derived from ESCs is not very high (approximately 80–90%) for transplantation purposes, and *in vitro* culture of these cells is slow and tedious^14^,^15^,^16^. Several studies have shown that CNS remyelination is closely linked to the acute inflammatory phase of disease, whereas in the chronic stage, remyelination strategies fail, regardless of whether they involve inducing endogenous repair or cell transplantation-based therapy^2^,^17^,^18^. Thus, novel therapeutic approaches are needed to promote tissue repair.

Recent studies have demonstrated that the posttranscriptional control of gene expression by microRNAs (miRNAs) plays a critical role in oligodendrocyte development. Several microRNAs are induced concurrent with oligodendrocyte differentiation, including miR-219, miR-338, miR-138, miR-29, and miR-23^19^,^20^,^21^,^22^,^23^. Among the most abundant miRNAs in mature oligodendrocytes, miR-219 is necessary to promote oligodendrocyte differentiation, in part by directly targeting negative regulators of oligodendrocyte development such as PDGFRα, Sox6 and Hes5, all of which normally help promote OPC proliferation. Additionally, miR-219 downregulates NeuroD1 to suppress neuronal differentiation and shifts the transition of NSCs toward the oligodendrocyte lineage^19^. A recent study reported that human endometrial-derived stromal cells (EnSCs) can be programmed into pre-oligodendrocyte cells via the overexpression of miR-219^24^. Young and environmentally enriched exosomes deliver functional miR-219, which promotes oligodendrocyte differentiation and enhances myelination in aging rats^25^.

The evidence indicates that miR-219 plays a critical role in enabling the rapid transition from proliferating OPCs or NSCs to myelinating oligodendrocytes. The present study aims to give new insights into the role of miR-219 in the differentiation of mESCs into oligodendrocyte lineage cells *in vitro* and in an *in vivo* model of chronic experimental demyelination. We performed oligodendrocyte lineage cell differentiation studies using miR-219-overexpressing mESCs. To determine whether miR-219-overexpressing OPC grafts promote remyelination more efficiently *in vivo*, we transplanted OPCs derived from miR219-mESCs into cuprizone-induced chronically demyelinated mouse brains. The data demonstrate for the first time that miR-219 rapidly transforms mESCs into oligodendrocyte lineage cells *in vitro* and that miR219-OPCs transplantation not only promotes remyelination and improves cognitive function but also enhances host endogenous NPC proliferation following chronic demyelination. These data are a valuable resource with which to understand the potential therapeutic role of miR-219 in demyelinating diseases and to better manipulate ESCs for cell replacement therapy.

## Results

### miR219-mESCs maintained pluripotency

**Figure 1:**
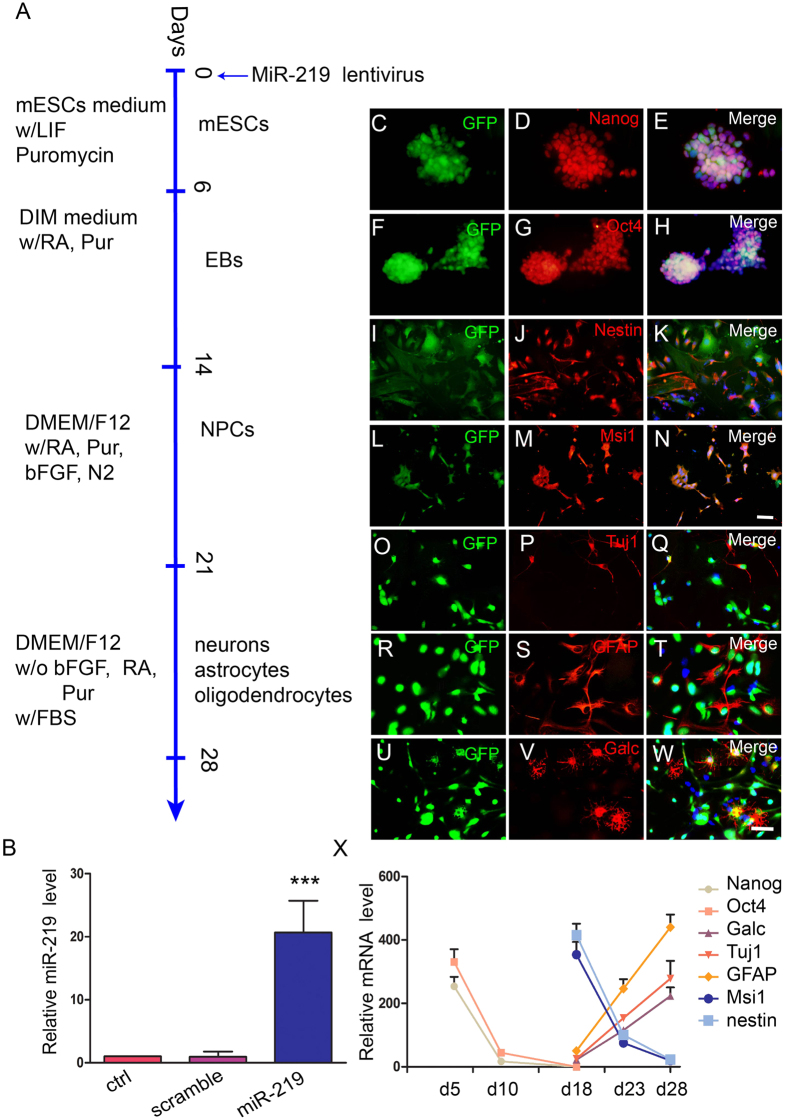
MiR-219-overexpressed mESCs are pluripotent. (**A**) A schematic protocol for directed differentiation of miR219-mESCs. Mouse ESCs were infected by miR-219 lentivirus at day 0. Twenty-four hours later, infected cells were selected under puromycin and grown in mESC medium without bFGF for 5 days, then switched to DIM medium supplemented with Pur and RA for 8 days to generate EBs. To induce NPCs, EBs were plated onto laminin/poly-ornithine-coated plates and cultured in DMEM/F12 supplemented with bFGF, RA, Pur and N2 for 7 days. On day 21, the medium was replaced with DMEM/F12 medium with 1% FBS but without bFGF, RA or Pur. (**B**) Relative miR-219 levels were detected by RT-PCR. The values shown are mean ± s.e.m. (n = 3), ***P < *0.001*. (**C–H**) Undifferentiated miR219-mESC colonies express the pluripotency markers Nanog and Oct4. (**I–N**) miR219-mESC-derived GFP-positive NPCs overlap with nestin and musashi1 (Msi1). (**O–W**) *In vitro* terminal differentiation of miR219-mESCs into neurons, astrocytes, and oligodendrocytes was identified by Tuj1, GFAP and Galc. From C to N, scar bars = 50 μm (in Fig. 1N). From O to W, scale bars = 50 μm (in Fig. 1W). (X)) The mRNA levels of Nanog, Oct4, Galc, Tuj1, GFAP, Msi1 and nestin were detected by RT-PCR.

To observe the role of miR-219 in mESC differentiation, we followed the differentiation protocol shown in Fig. 1A. At 24 h, mESCs infected by miR-219 lentivirus were selected under 4 ng/ml puromycin. After the puromycin-resistant mESCs were passaged more than 10 times, both the miR-219 vector lentivirus-infected and the scramble vector lentivirus-infected mESCs presented as GFP-positive. RT-qPCR showed that miR-219 was successfully overexpressed in mESCs and that the relative miR-219 level in miR219-mESCs was 22.4 times higher than in the scramble group at 3 days (Fig. 1B, *p* = *0.0001*). The selected cells were maintained in an undifferentiated proliferative state for 5 days; they were then switched to DIM medium for 8 days to form EBs. As expected, undifferentiated miR219-mESC colonies expressed strong GFP fluorescence and were positive for the mESC markers Nanog (Fig. 1C–E) and Oct4 (Fig. 1F–H). EBs were trypsinized and plated in N2 medium supplemented with bFGF for 7 days, as shown in Fig. 1I–N, and GFP-positive cells overlapped with the NPC markers nestin (Fig. 1I–K) and musashi1 (Fig. 1L–N). Upon exposure to terminal differentiation medium for 7 days, GFP/Tuj1 double-positive neurons (Fig. 1O–Q), GFP/GFAP double-positive astrocytes (Fig. 1R–T) and GFP/Galc double-positive oligodendrocytes (Fig. 1U–W), respectively, formed from the NPCs. The mRNA expression of Nanog, Oct4, nestin, musashi1, Tuj1, GFAP and Galc were consistent with the results of immunocytochemistry (Fig. 1X). Thus, we verified that miR-219 overexpression did not affect the properties of mESCs, which maintained pluripotency and were able to give rise to the three main CNS cell types upon differentiation.

### MiR-219 accelerated the differentiation of mESCs along an oligodendrocyte linage

**Figure 2:**
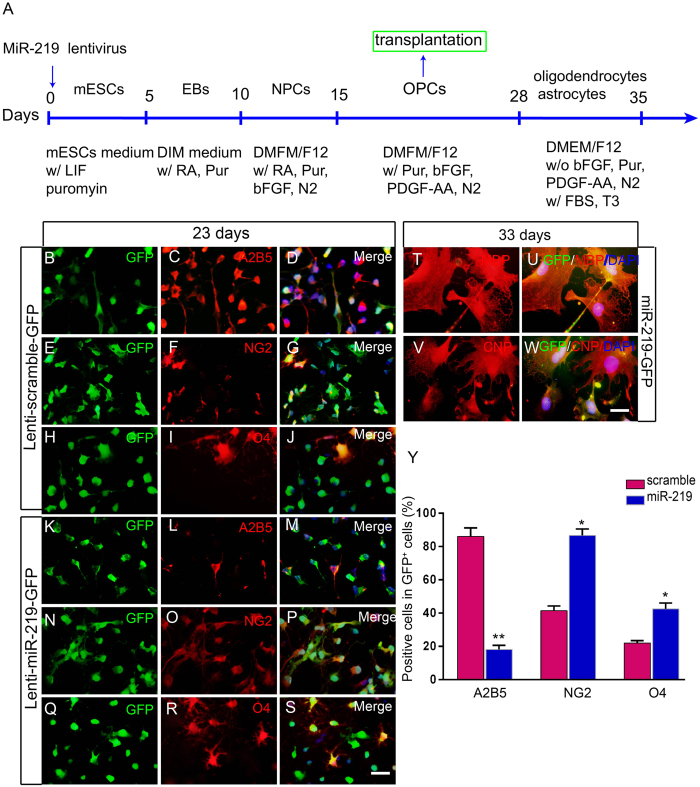
MiR-219 accelerates mESC differentiation into an oligodendrocyte lineage. (**A**) A schematic protocol for the directed differentiation of miR219-mESCs into an oligodendrocyte linage. To generate OPCs, on day 15, NPCs were derived from mESCs grown for up to 28 days in DMEM/F12 supplemented with 10 ng/mL bFGF, 10 ng/mL PDGF-AA, 1% N2, and 2% KSR. Fluorescence immunocytochemistry imaging revealed that most of the GFP-positive cells in the scramble group were also A2B5 positive (**B–D**); a few were NG2 (**E–G**) and O4 (**H–J**) positive at 23 days *in vitro*. In the miR-219 group, GFP-positive cells expressed weak A2B5 (**K–M**) and strong NG2 (**N–P**) and O4 (**Q–S**) fluorescence intensity. Nuclei were stained with DAPI (blue). For oligodendrocyte maturation, miR219-OPCs were cultured in DMEM/F12 with 10% FBS for 7 days. Images show that OPCs differentiated into MBP- (**T–U**) and CNP- (**V–W**) positive mature oligodendrocytes. The percentages of A2B5-, NG2- and O4-positive cells in GFP-positive cells were counted (**X**). The data are presented as the mean ± s.e.m. *P < 0.05, **P < *0.001*; n = 3 for each group. Scale bars: S = 50 μm; W = 20 μm.

Although miR219-mESCs maintained pluripotency, it is not clear whether miR-219 influences the differentiation of mESCs into the oligodendrocyte lineage. To explore this question, we induced miR219-mESCs using the protocol shown in Fig. 2A to test the expression of NPC or OPC marker A2B5, the pre-oligodendrocyte marker NG2 and the immature oligodendrocyte marker O4. At 20 days, both scramble and miR-219 groups expressed strong A2B5 and NG2 fluorescence, no O4-positive cells were found in the scramble group, and a few O4-positive cells were detected in the miR-219 group (data not shown). Three days later (23 days), the expression of A2B5 (Fig. 2B–D) was still strong in the scramble group, and NG2 (Fig. 2E–G) and O4 (Fig. 2H–J) were detected with weak fluorescence. In the miR-219 group, the expression of A2B5 was decreased (Fig. 2K–M), whereas that of NG2 (Fig. 2N–P) and O4 (Fig. 2Q–S) was further increased with strong fluorescence. This overlapping expression of markers for early and late OPCs indicates cells at different developmental stages. The number of A2B5-positive cells was 4.78 times lower in the miR-219 group (Fig. 2X; 18.15 ± 3.82 versus scramble 86.05 ± 7.14), whereas the numbers of NG2- and O4-positive cells significantly increased in the miR-219 group compared to the scramble group (Fig. 2X; NG2 86.60 ± 6.93 versus scramble 41.42 ± 5.43; O4 42.51 ± 5.88 versus scramble 22.03 ± 3.57), suggesting that overexpression of miR-219 accelerated the differentiation of mESCs into OPCs.

Mature oligodendrocyte markers MBP and CNPase probed using immunocytochemistry showed that miR219-mESC derived OPCs (miR219-OPCs) were able to differentiate into mature oligodendrocytes *in vitro*, almost all oligodendrocytes that differentiated from miR219-OPCs were MBP positive (Fig. 2T–U) and CNPase positive (Fig. 2V–W).

### MiR219-OPCs were associated with spinal axons in co-culture

**Figure 3:**
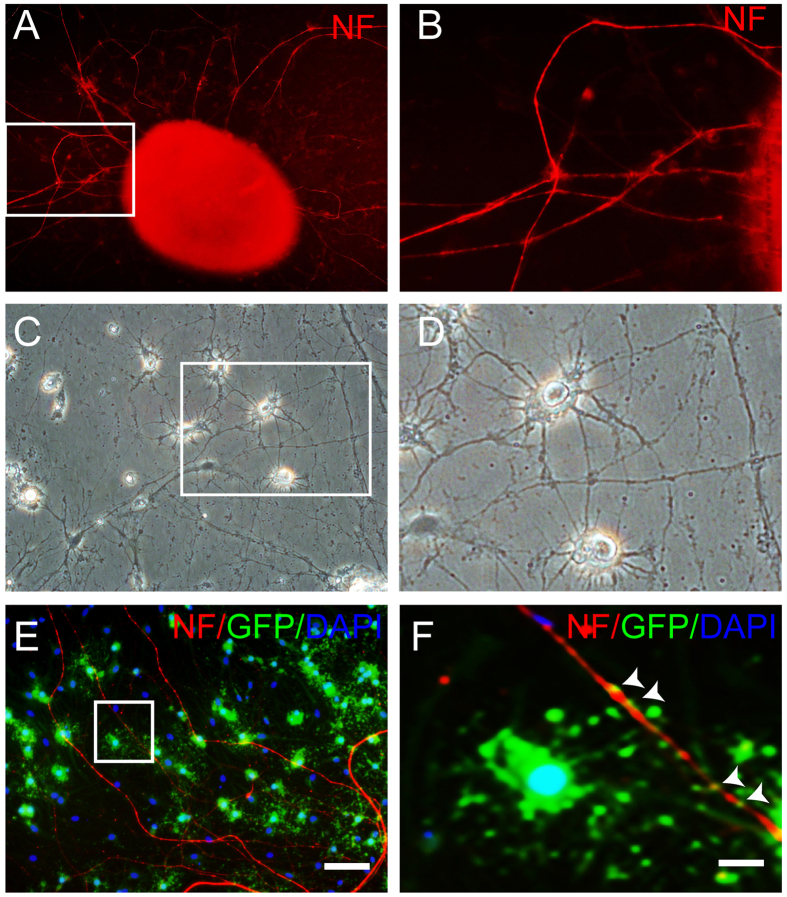
MiR219-OPCs are associated with spinal axons in co-culture. (**A,B**) NF-200 immunocytostaining shows neurites extending from spinal cord explants. High-magnification images of the box in A are shown in B. (**C,D**) Phase contrast micrographs show miR219-OPCs-derived cells exhibiting multipolar morphology and interacting with the neurites from spinal cord explants. The boxed area in C is presented in higher magnification in D. (**E,F**) After 2 weeks of co-culture, the processes of some GFP-positive oligodendrocytes have touched and elongated along the NF-200-positive neurites. The white arrows in image F and the high-magnification images of the box in image E indicate that GFP-positive oligodendrocytes made close contact with the neurites. Scale bars: A, C, E = 50 μm; B, D, F = 10 μm.

The behavior of miR219-OPCs *in vitro* was observed by OPCs and spinal cord explants co-culture. Before co-culture, spinal cord explants were plated onto coverslips coated with 2% matrigel. Three days later, the neurites extended radially from the explants, but their somas remained in the explants; immunostaining showed that the neurites were positive for the neuron axonal marker NF-200 (Fig. 3A,B). Two weeks after co-culture, phase contrast micrographs showed that the oligodendrocytes that differentiated from miR219-OPCs exhibited multipolar morphology and attached to the neurites growing out from the spinal cord explants (Fig. 3C,D). Immunostaining confirmed that the processes of some GFP^−^ positive oligodendrocytes had touched and elongated along the neurites (Fig. 3E,F), showing the association of the OPCs with spinal axons in co-culture.

### Survival and differentiation of miR219-OPCs after transplantation

**Figure 4:**
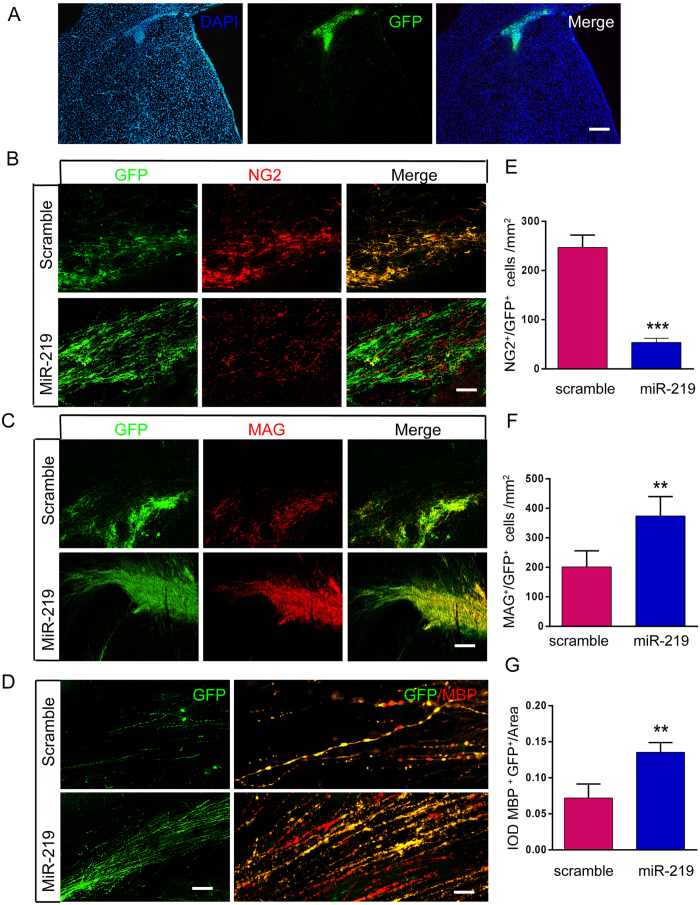
MiR-219 promotes the survival and differentiation of OPCs after transplantation in cuprizone-induced demyelinated mice. (**A**) Representative images showing that surviving OPCs were identified at the injection site as GFP-positive cells 1 week after transplantation. (**B**) GFP-positive cells were co-labeled with the OPC marker NG2 in the corpus callosum 2 weeks after transplantation, representative image of n = 3. (**C**) At 4 weeks post-transplantation, a large fraction of GFP-positive cells in both the scramble and miR-219 groups co-expressed the oligodendrocyte marker MAG, representative image of n = 3. (**D**) MBP was observed when the OPC grafts survived for up to 8 weeks, representative image of n = 3. (**E–G**) Quantification of the number of NG2/GFP or MAG/GFP double positive cells, as well as the IOD of MBP/GFP double positive cells in the corpus callosum. The data are presented as the mean ± s.e.m. **P < 0.01, ***P < *0.001*; Scale bars: A = 100 μm; B, C = 50 μm; D = 20 μm. IOD, integral optical density.

To assess whether miR-219 promotes a more effective transplant engraftment, OPCs derived from mESCs were injected by stereotaxic surgery into the corpus callosum of cuprizone-induced demyelinated mice. The injection site (Fig. 4A) was indicated by the presence of GFP-positive cells at 1 week after transplantation, and the survival and differentiation of OPCs were examined by fluorescent immunohistochemistry at 2, 4 and 8 weeks after transplantation.

We found that the majority of surviving GFP-positive cells remained NG2-positive with undifferentiated shapes in the scramble group at 2 weeks. In contrast, a minority of GFP-positive cells became negative for NG2 in the miR-219 group (Fig. 4B,E; 246.90 ± 25.30 versus scramble 53.63 ± 8.43/mm^2^), although the majority were MAG positive (data not shown). The data suggested that fewer NG2-positive OPCs had differentiated into immature or mature oligodendrocytes in the scramble group; however, miR-219 likely promoted the differentiation of OPC grafts *in vivo*.

At 4 weeks post-transplantation, a large fraction of GFP-positive cells in both groups co-expressed MAG, which is essential for the initiation of myelination. The number of MAG/GFP double positive cells in the miR-219 group was significantly increased compared to the scramble group (Fig. 4F; 373.83 ± 26.94 versus scramble 201.28 ± 20.73). Moreover, MAG/GFP double positive cells in the miR-219 group acquired a more mature oligodendrocyte shape (Fig. 4C), indicating that these cells had differentiated into oligodendrocytes, and miR-219 promoted the earlier maturation of oligodendrocytes, in accordance with observations from *in vitro* experiments.

Another structural protein, MBP, which plays a vital role in myelin compaction and thickening in the CNS, was observed for up to 8 weeks. MBP/GFP double positive cells seemed to wrap around neurites, and the bundles of MBP/GFP double positive nerve fibers were denser in the miR-219 group than in the scramble group (Fig. 4D). Mice in the miR-219 group had significantly increased MBP/GFP double positive IOD values compared with scramble group mice, as shown in Fig. 4G (3368.6 ± 321.2 versus scramble 1812.4 ± 219.1).

We also detected the activation of astrocytes and microglia (data not shown); a slight increase was detected for GFAP and Iba-1 expression in OPC transplanted mice compared with control mice, but an obvious decrease in GFAP and Iba-1 expression was observed in miR219-OPC transplanted mice compared with scramble mice. No tumor formation was observed in the mouse brains. Our results demonstrated that OPCs, which were generated *in vitro* from miR219-mESCs, can survive transplantation and effectively promote oligodendrocyte differentiation *in vivo*.

### MiR-219 promoted remyelination *in vivo*

The extent of demyelination and remyelination during cuprizone exposure and recovery was further analyzed by LFB staining. At 12 weeks of cuprizone exposure, all myelin sheaths had been cleared in the corpus callosum of exposed mice (Supplementary Fig. 1B), whereas in the control group, the myelin sheaths were complete (Supplementary Fig. 1A), which was a significant difference between the groups (Supplementary Fig. 1F, *p* < *0.0001*). Cuprizone-induced demyelination was followed by a recovery phase on a normal diet and HBSS injection for 8 weeks (vehicle group); remyelination was observed but was strongly limited. The amount of re-expressed myelin, as judged by LFB staining, was approximately 25.27% (Supplementary Fig. 1C,F). Transplantation of OPCs promoted remyelination, as shown in Supplementary Fig. 1D–F. The myelin scores significantly increased both in the scramble group and the miR-219 group compared with the vehicle group (scramble versus vehicle *p* < 0.05; miR-219 versus vehicle *p* < 0.01). Moreover, the myelin score was higher in the miR-219 group than in the scramble group (*p* < 0.05).

**Figure 5:**
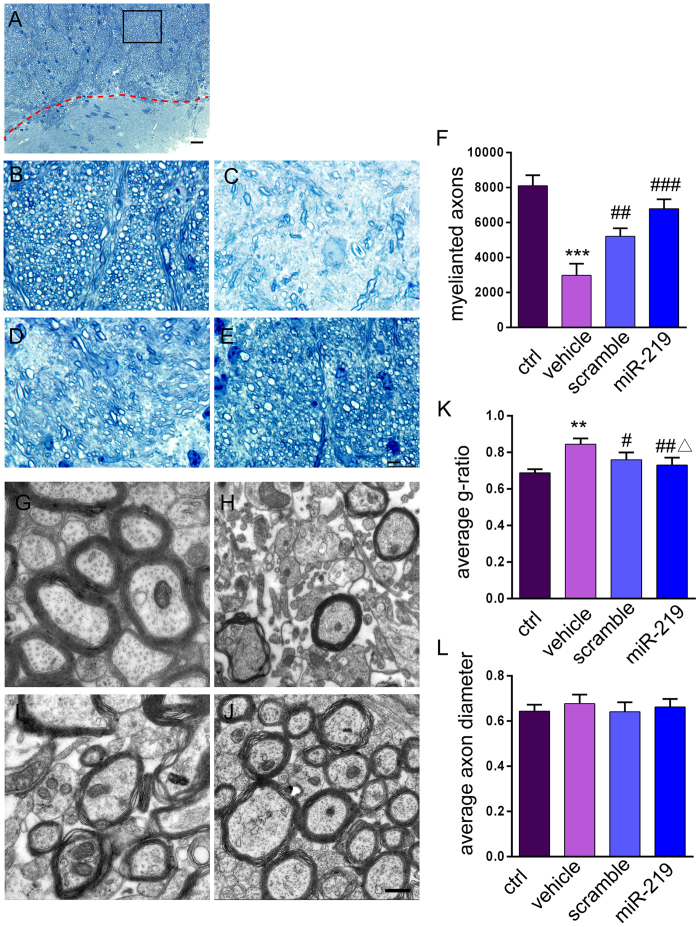
Enhanced remyelination in miR219-OPC-transplanted mice. Toluidine blue staining of semi-thin resin sections of the corpus callosum in control mice (**A,B**), mice fed a cuprizone diet for 12 weeks followed by a normal diet for 8 weeks, treated with vehicle (**C**), engrafted with scramble-OPCs (**D**) and engrafted with miR219-OPCs (**E**). Dotted lines in A denote the borders of the corpus callosum and the striatum. The boxed image in A is shown in higher magnification in B. (**F**) The bar graph indicates the mean ± s.e.m of the number of myelinated axons (n = 4 per condition). Representative electron microscopic images of cross-sections of the corpus callosum of control mice (**G**), vehicle treated mice (**H**), scramble-OPCs transplanted mice (**I**) and miR219-OPC transplanted mice (**J**). The *g* ratio (axon diameter/fiber diameter) calculation (n = 4 per group, with 25–30 axons measured per group) significantly decreased in the scramble- and miR219-OPCs-transplanted groups, indicating improved myelin thickness (**K**). (**L**) Axon diameter was not significantly different in different samples (n = 4 per group, with 25–30 axons measured per group). All values are expressed as the mean ± s.e.m. **P < *0.01,* ***P < *0.001* versus control group; ^*#*^*P* < *0.05,*^*###*^*P* < *0.001* versus vehicle group; ^Δ^*P* < *0.05* versus scramble group. Scale bars: A = 20 μm, B-E = 10 μm and G-J = 500 nm.

Toluidine blue staining of semi-thin sections of corpus callosum showed a significant decrease in the number of myelinated axons in mice with 12 weeks of a cuprizone diet followed by 8 weeks of a normal diet compared with control mice (Fig. 5A,B,F; *p* = *0.0002*). In mice engrafted with OPCs, significantly enhanced numbers of myelinated axons were detected compared with vehicle group mice (Fig. 5D,E,F; scramble *vs.* vehicle *p* = *0.0193,* miR219 *vs.* vehicle *p* = *0.0009*). Interestingly, the number of myelinated axons in the miR219-OPC transplanted mice was greater than in the scramble-OPC transplanted mice (Fig. 5F; *p* = *0.0331*), suggesting that miR-219 might be beneficial for remyelination.

Next, ultrastructure analyses were performed to show myelinated axons in the corpus callosum. Regular myelin sheaths were observed in mice fed normal chow (Fig. 5G). The mice showed the complete demyelination of axons after 12 weeks of cuprizone feeding (data not shown). After a 12-week cuprizone diet followed by 8 weeks of a normal diet, although some axons were remyelinated, the majority remained severely demyelinated. A number of large swollen axons with broken myelin sheaths was found, and the myelin sheaths broke down and compact layers split apart irregularly (Fig. 5H). In contrast, improved myelination was found in the corpus callosum of OPC-transplanted mice. However, some myelin sheaths still presented altered myelin structures, including hypomyelination, splits and vacuolated formations, especially in the scramble group. Less disturbed myelin sheaths and more compact and thicker myelin sheaths were found in the miR-219 group (Fig. 5I–J). Due to myelin thickness, we observed a significant increase in the *g-*ratio of vehicle samples compared to the control samples (Fig. 5K, *p* = *0.0013*), and a significant decrease in the *g-*ratio of miR219-OPC transplanted samples compared to vehicle samples (Fig. 5K, *P* = *0.0100*) and scramble samples (*p* = *0.0219*). A further decrease in the *g-*ratio in the miR219 group was observed compared to the scramble group (Fig. 5K, *P* = *0.0402*). The differences in the average axon diameter were not significant among the groups (Fig. 5L). These ultrastructural examinations revealed that miR219-OPC transplantation promoted remyelination more efficiently than scramble-OPC transplantation.

### Transplanted OPCs are involved in the induction of endogenous NPC proliferation

The SVZ contains four main cell types defined by their morphology, ultrastructure, and molecular markers: migrating neuroblasts (type A cells), astrocytes (type B cells), proliferative precursors (type C cells) and ependymal cells (type E cells). A subpopulation of B cells are the primary NPCs, which are converted to transient amplifying type C cells that generate neuroblasts and glial cells^26^,^27^.

**Figure 6:**
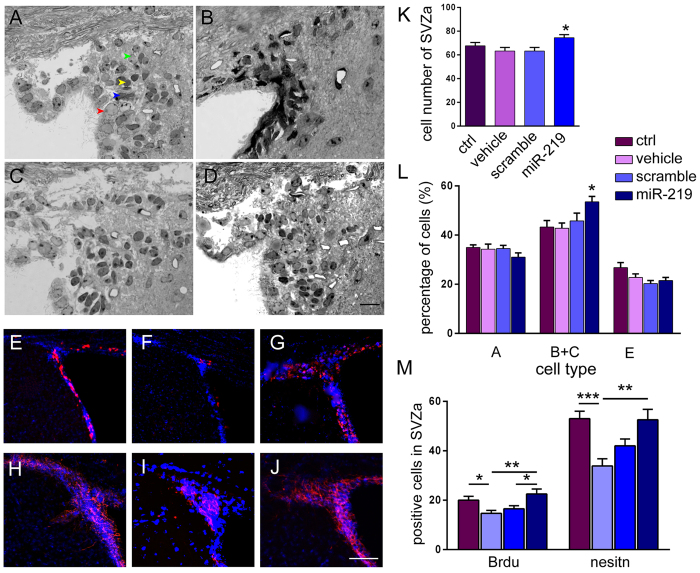
OPC transplantation enhances the proliferation of host SVZa cells in cuprizone-induced chronic demyelination lesions. Mice were sacrificed and the cell types in the SVZa were assessed after transplantation 8 weeks. Semi-thin sections stained with toluidine blue (we converted the colour pictures into grey) show the cells in the SVZa in control (**A**), vehicle (**B**), scramble (**C**) and miR-219 groups of mice (**D**). Arrows in Figure A represent the various cell types: type E (red), type B (blue), type A (yellow) and type C (green). The bar graphs (**K**,**L**) indicate the total cell numbers and the different cell types in the SVZa (AOI, 600 μm × 450 μm). BrdU- (**E–G**) and nestin- (**H–J**) positive cells were also detected in the SVZa by immunofluorescence. Representative images are the same fields from three different conditions. The numbers of BrdU- and nestin-positive cells increased in the SVZa of miR219-OPC-transplanted mice, compared with vehicle treated mice (**M**) (AOI, 200 μm × 100 μm). All values are expressed as the mean ± s.e.m. (n = 4 per condition for semi-thin sections, n = 3 per condition for immunostaining). *P < *0.05,* **P < *0.01* and ***P < *0.001*. Scale bars: A–D = 10 μm and E-J = 100 μm.

NPC transplantation has been reported to enhance host-derived myelin regeneration following chronic demyelination^4^. We examined the effect of OPC transplantation on the proliferation of SVZa cells *in vivo*. Semi-thin sections of SVZa showed no significant differences in total cell number among the control, vehicle and scramble groups (Fig. 6A,B,C,K). However, the total cell number in the SVZa was significantly increased in mice engrafted with miR219-OPCs compared with the vehicle group (Fig. 6D,K; *p* = *0.0136*). We further analyzed the numbers of various cell types. The groups did not significantly differ in the numbers of type A or E cells. However, the total numbers of proliferating type B and type C cells in the SVZa were significantly increased in mice engrafted with miR219-OPCs compared with the vehicle group (Fig. 6L; *p* = *0.0151*). The morphology of the four main cell types was further identified by ultrastructural examinations (Supplementary Fig. 2). The numbers of BrdU- and nestin-positive cells were detected in the SVZa by immunofluorescence (Fig. 6E–J). Cuprizone induced a significant decrease in BrdU- and nestin-positive cells compared with control mice (BrdU *p* = *0.0224;* nestin *p* = *0.0003*). However, miR219-OPCs grafts exhibited consistently elevated numbers of BrdU- and nestin-positive cells (Fig. 6M, BrdU *p* = *0.0075;* nestin *p* = *0.0021*). The numbers of BrdU-positive cells were significantly increased in the miR219 group compared to the scramble group (*p* = *0.0300*).

### OPC transplantation improves cognitive deficits in cuprizone-treated mice

#### Step-through passive avoidance task

**Figure 7:**
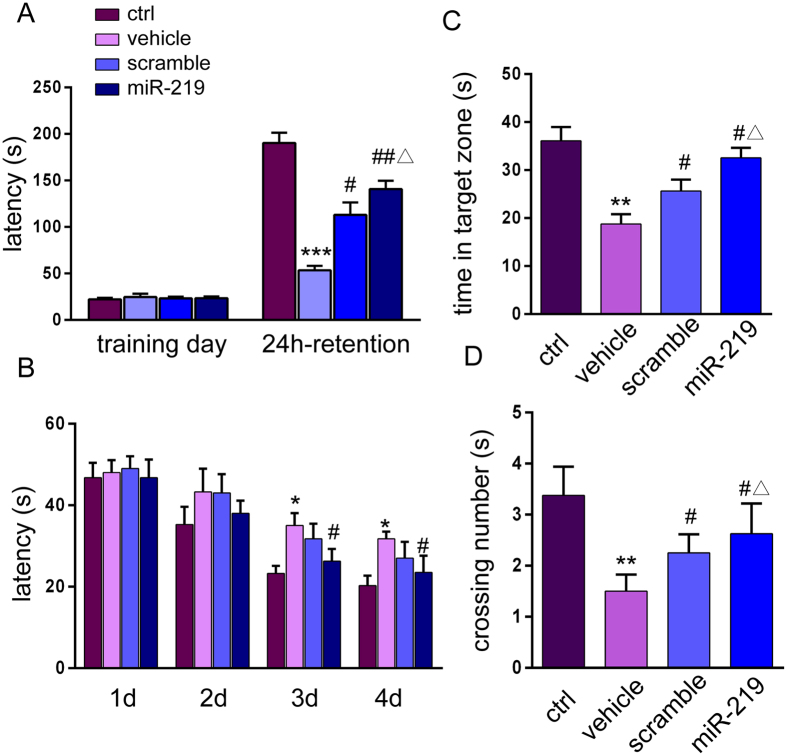
OPC transplantation reverses cognitive deficits in cuprizone-induced demyelinated mice as shown by the step-through passive avoidance task and the MWM test. (**A**) Step-through passive avoidance task. (**B**) Mean latency in the MWM hidden platform test. (**C**) Comparison of the time spent in the target quadrant on day 5 (where the platform was located during hidden platform training). (**D**) The number of crossings over the exact location of the former platform on day 5. The data are from 8 mice/group. All values are expressed as the mean ± s.e.m. *p < *0.05*, **p < *0.01*, ***p < *0.001* versus control group; ^*#*^*p* < *0.0*5, ^*##*^*p* < *0.01* versus vehicle group, ^Δ^*P* < *0.05* versus scramble group.

In the acquisition trial, the initial latency did not differ among the four groups [non-significant versus ctrl group] (Fig. 7A). The step-through latency in the 24-h retention trial was significantly decreased in cuprizone-treated mice (vehicle *vs.* ctrl *p *=* 0.0001*). Analysis showed that OPC transplantation significantly inhibited the amnesic effect of the cuprizone diet on latency time for the step-through trial. The latency time was markedly increased in mice engrafted with scramble-OPCs or miR219-OPCs (scramble *vs.* vehicle group *p *=* 0.021*; miR-219 *vs.* vehicle *p *=* 0.004*). The latency time was further enhanced in the miR-219 group compared to the scramble group (*p *=* 0.034*).

#### Morris water maze task

Statistical analysis of the escape latency in the MWM task was performed using two-way ANOVA for repeated measures with day and treatment as the sources of variation (Fig. 7B). The results showed that mice fed the cuprizone diet had longer escape latencies than did control mice (which did not receive cuprizone) on Days 3 and 4 (*p *=* 0.0260*). A comparison between the vehicle group and the miR219 group showed that the transplantation of miR219-OPCs decreased the escape latency of mice fed the cuprizone diet (*p *=* 0.0331*), whereas the scramble group and the vehicle group had no significant differences in their spatial learning or memory abilities. All of the mice had the same levels of performance at the start of the experiment (no significant individual effects were observed for the first four trials of Day 1).

On the fifth testing day, the platform was removed, and the probe test was performed. The vehicle group spent less time swimming in the target quadrant (where the platform was located) than did the control group (*p *=* 0.0063*), whereas the scramble and miR-219 groups spent more time swimming in the target quadrant than did the vehicle group (scramble *vs.* vehicle *p *=* 0.0310*, miR-219 *vs.* vehicle *p *=* 0.0205*, Fig. 7C). The results also show a significant increase in the swimming time of miR-219 mice compared with scramble mice (*p *=* 0.040*, Fig. 7C).

Similar results were obtained for the mean number of times the animals crossed the place where the platform was located during training. The vehicle group mice crossed over the platform less frequently than the control mice did (*p *=* 0.0047*). The mean number of times significantly increased in the scramble and the miR-219 groups compared with the vehicle group (Fig. 7D, scramble *vs.* vehicle *p *=* 0.0330*, miR-219 *vs.* vehicle *p *=* 0.018*). A difference was also observed in the number of crossings of the scramble group and the miR-219 group (*p *=* 0.044*, Fig. 7D).

## Discussion

Current studies using experimental animal models of demyelination are aimed at discovering therapeutic strategies to replace myelin under circumstances in which endogenous remyelination is unsuccessful. Here, we show for the first time that miR-219 rapidly transforms mESCs into oligodendrocyte lineage cells *in vitro* and that miR219-OPC transplantation not only promotes remyelination and improves cognitive function but also enhances host endogenous NPC proliferation in a chronic demyelination mouse model, thus suggesting a possible therapy for demyelinating diseases.

Protocols for the differentiation of ESCs to OPCs have been developed according to developmental principles^28^,^29^. However, the efficiencies of ESC differentiation into OPCs are inconsistent when the procedure is performed by different laboratories. It is necessary to purify ESC-derived OPCs and improve the survival and differentiation of myelinating oligodendrocytes to generate a safe population for use in regenerative therapy^30^. Genetic modifications have been made to ESCs to induce the expression of specific classes of transcription or growth factors to enhance OPC differentiation^29^,^31^,^32^. Furthermore, miRNAs can be tightly integrated into molecular pathways and cooperate to control the fate of ESCs. mESCs that lack miRNAs due to Dicer1 or Dgcr8 deficiency do not proliferate well and display severe differentiation defects^33^. Additionally, individual miRNA functions have been revealed in ESCs^34^,^35^. However, it is unknown whether miR-219 controls the oligodendrocyte lineage commitment of ESCs. Therefore, we explored the potential of miR-219 to promote ESC differentiation into an oligodendroglial lineage in vitro and the utility of miR219-OPCs as a cell replacement neuroprotective therapy.

We initially tested the effect of miR-219 on mESC differentiation without differentiating cocktails *in vitro*. Our results demonstrated that miR-219 overexpression did not affect the properties of mESCs, which maintained pluripotency and could be induced to form EBs and NPCs. Upon terminal differentiation, the three main types of neural cells (astrocytes, oligodendrocytes and neurons) in the CNS were obtained from NPCs, as previously described by others^36^. Gene expression analyses for markers of ESCs, NPCs and terminally differentiated cells further confirmed the results of immunocytochemistry. Moreover, the expression of A2B5, NG2 and O4 was not detected (data not shown) when these cells were maintained in mESC medium, which suggests that miR-219 might be a non-sufficient factor, not a switch, for mESC differentiation at an early stage.

Previous protocols have described the generation of an oligodendroglial lineage from mESCs at specific points in time^14^. Here, the capacity of miR219-mESCs to induce the differentiation of an oligodendrocyte linage was investigated by A2B5 and NG2 (OPC markers) and O4 (immature oligodendrocyte marker) immunocytostaining following the general protocol described above. For the first time, we found that the overexpression of miR-219 accelerated the differentiation of mESCs into OPCs. In differential medium containing FBS and T3, miR219-OPCs developed into mature oligodendrocytes by expressing CNPase and MBP, which is critical in the initial steps leading up to myelination. However, whether these glial cells shift to further axonal association and ensheathment to form myelin remains to be determined.

The *in vitro* models developed to investigate the growth and myelination of axons in the CNS, such as dorsal root ganglion (DRG) neuron–oligodendrocyte co-culture^37^, CNS neuron-oligodendrocyte co-culture^38^, and spinal cord explant-oligodendrocyte co-culture^39^, have revealed the mechanisms that underlie the interaction between neurons and myelinating cells. In this study, we used a spinal cord explant-oligodendrocyte co-culture model to observe the association of miR219-OPCs with spinal axons because without the interference of non-GFP somas, the interaction between GFP-oligodendrocytes and neurites could be observed clearly and dynamically without immunostaining. Two weeks after co-culture, the oligodendrocytes that differentiated from miR219-OPCs exhibited multipolar morphology, and the processes of some GFP-positive oligodendrocytes touched and elongated along the neurites, thus suggesting the association of OPCs with spinal axons. Of course, it is not sufficient to show only GFP and draw conclusions regarding myelination capacity here. Further examination with Sudan black, Caspr, or TEM is necessary to draw any meaningful conclusions.

No animal model completely recapitulates the complex pathomechanisms of human MS; animal models can mimic only parts of MS pathology. Cuprizone is a copper chelating reagent that, when added as a supplement to normal rodent chow, directly or indirectly causes oligodendroglial cell death with subsequent demyelination. Spontaneous remyelination is rapid and robust after acute demyelination [5 weeks of 0.2% (w/w) cuprizone administration]^40^,^41^. However, after a long exposure to cuprizone (12 weeks), known as chronic demyelination, spontaneous myelin regeneration no longer occurs for at least up to 9 weeks^42^,^43^. Thus, the cuprizone model has gained attention and acceptance in recent years because it is particularly suitable for investigating remyelination without the possible interference of peripheral immune cells such as macrophages and lymphocytes^44^,^45^. Here, we took advantage of the fact that chronic cuprizone exposure in C57BL/6 mice results in a less than 30% rate of spontaneous remyelination to study the role of miR219-OPCs after transplantation.

The transplantation of OPCs promotes remyelination and ameliorates experimental models of demyelination in the acute phase aside from remyelination, via neuroprotection, the suppression of inflammation, the promotion of axonal regeneration, and/or homeostatic maintenance^13^,^46^,^47^,^48^. The administration of thyroid hormone has been found to promote myelin repair under conditions of severe and chronic demyelination^42^. However, it is unknown whether ESCs-derived OPCs can promote remyelination in chronically demyelinated lesions, in which the pro-remyelinating effects of inflammation are diminished^49^. To this end, upon the transplantation of OPCs into chronically demyelinated mice, scramble-OPCs remained NG2 positive, and by 4 weeks, a minority of these cells began to differentiate into MAG-positive oligodendrocytes. By 8 weeks, a few MBP/GFP double positive nerve fiber bundles were detected in the corpus callosum. ESC-derived OPC transplantation faces huge challenges in chronic demyelination diseases because of the lack of a microenvironment that promotes OPC survival and differentiation^50^. Excitingly, miR219-OPCs grafts effectively survived and promoted mature oligodendrocyte differentiation *in vivo*. Moreover, ultrastructure analyses confirmed these observations by showing that the number of myelinated axons increased and the *g-*ratio decreased after transplantation. There is accumulating evidence that OPC transplantation-mediated neuroprotection occurs through a variety of mechanisms, including reducing endogenous neuronal and oligodendrocyte cell loss, stimulating the proliferation of endogenous progenitors, remyelinating the brain and providing neurotrophic support independent of directed differentiation^48^,^51^,^52^. Recently, Chen *et al*. suggested that transplanted OPCs not only survived and formed a myelin sheath but also stimulated BDNF and Bcl-2 expression and the proliferation of NSCs while inhibiting hypoxic-ischemic-induced neuronal apoptosis^52^. miR219-OPCs promote remyelination via direct differentiation and the formation of new oligodendrocytes. Otherwise, miR219-OPCs may benefit the environment by secreting neurotrophic factors or inhibiting inflammation.

Although the presence of OPCs throughout the CNS has been observed in MS patients, it is still undetermined to what extent OPC/NPC death occurs in MS lesions^53^, with subsequent reduction of the endogenous pools of these cells. Another important and unresolved question is whether the size of this pool directly impacts the efficiency of the repair process^54^. The SVZ and the parenchyma, which includes the white matter itself, are the two major sources of OPCs in the adult brain. Upon chronic cuprizone administration (12–16 weeks), newly generated oligodendrocytes progressively undergo apoptosis, and the pool of OPCs becomes diminished^55^. Armstrong *et al*. also reported that the density of PDGFαR+ cells was significantly increased during acute demyelination but decreased to untreated levels during chronic cuprizone administration. The density of Ki-67+ cells also declined during the recovery phase after 12 weeks of cuprizone^56^. Our data show that the number of proliferating OPCs decreased in chronic demyelinated mice, which is consistent with previous reports. However, we also found an increase in the total numbers of various cell types in the SVZa following miR219-OPC transplantation. miR219-OPC transplantation induced an increase in the number of proliferating NPCs, as confirmed by morphological analysis, BrdU labeling and nestin immunostaining. In the scramble group, there was a slight increase in NPCs but no obvious differences compared with the control and vehicle groups. In chronically demyelinated white matter, bone marrow-derived mesenchymal stromal cell (BM-MSC) transplantation activated endogenous OPCs originating from the SVZ and dentate gyrus and induced remyelination surrounding the grafted regions^57^. Here, we presume that endogenous NPCs may be beneficial to remyelination, but it is necessary to confirm the effects and mechanisms of these NPCs on remyelination.

Previous studies have shown that cuprizone-exposed mice display impaired spatial working memory^58^,^59^, decreased motor function^60^, and diminished social interaction^59^,^61^. Recently, Lewis rats fed with cuprizone exhibited demyelination and behavioral changes that share some similarities with white matter abnormalities observed in humans^62^. The abnormal behaviors, demonstrable demyelination, and oligodendrocyte loss observed in cuprizone-treated mice suggested a correlation between white matter damage and certain behavioral abnormalities. In this study, we first provided *in vivo* proof that OPC transplantation reverses cognitive deficits in cuprizone-induced demyelinated mice, as shown by the step-through passive avoidance task and the MWM test. The miR-219 group showed greater improvement on step-through test performance than the scramble group, suggesting that miR-219 facilitated cognitive functional improvement by promoting oligodendrocyte differentiation and remyelination. In addition, white matter repairment and endogenous NPCs proliferation might improve brain microenvironment which contributed to neurobehavioral function recovery.

In conclusion, remyelination strategies fail following demyelination because of obstacles to oligodendrocyte differentiation and maturation. Our findings demonstrate for the first time that miR-219 rapidly transforms mESCs into oligodendrocyte lineage cells *in vitro*. Moreover, miR219-OPC transplantation not only promotes remyelination and improves cognitive function but also enhances host endogenous NPC proliferation following chronic demyelination. These results support the potential therapeutic role of miR-219 in demyelinating diseases such as MS^62^.

## Methods

### Maintenance of mESCs

R1/E mESCs was obtained from Shanghai Institute of Biochemistry and Cell Biology, Chinese Academy of Sciences, and maintained as described in our previous studies^29^,^63^. In brief, mESCs were grown at an optimal density in gelatin (Sigma) coated dishes without feeder cells in Dulbecco’s modified Eagle’s medium (DMEM; GIBCO) supplemented with 10% fetal bovine serum (FBS; HyClone), 2 mM glutamine (Sigma), 1 mM sodium pyruvate (Sigma), 1× nonessential amino acids (NEAA; Invitrogen), 55 μM β-mercaptoethanol (Amresco), and 1 × 103 units/mL recombinant leukemia inhibitory factor (LIF; Sigma) in an environment containing 5% CO_2_ at 37 °C. The medium was changed every second day.

### Differentiation of mESCs

Based on a previously established protocol^64^, the neural differentiation of mESCs was initiated using the protocol shown in Fig. 1A. Briefly, to generate embryonic bodies (EBs), mESC colonies were trypsinized into single cells and suspended in differentiation medium (DIM) consisting of α-minimal essential medium (а-MEM; HyClone) supplemented with 20% knockout serum replacement (KSR; GIBCO), 1 mM sodium pyruvate (Sigma), 0.1 mM NEAA (Invitrogen), and 0.1 mM β-mercaptoethanol (Amresco). The suspended cells from mESCs were cultured for 4 days in DIM and then cultured for another 4 days in DIM medium supplemented with 1 μM purmorphamine (Pur; Sigma) and 200 nM retinoic acid (RA; Sigma) to form EBs.

To induce the formation of neural precursor cells (NPCs), EBs were disaggregated in TrypLE (Invitrogen) on day 9 and plated on 6-well plates coated with poly-L-ornithine (0.002%; Sigma)/fibronectin (10 μg/ml; Millipore) or poly-L-ornithine (0.002%)/laminin (50 μg/ml; Sigma), DMEM/F12 (GBICO) supplemented with 10 ng/ml basic fibroblast growth factor (bFGF; Invitrogen), 1 μM Pur (Sigma), 200 nM RA (Sigma) and 1% N2 medium (GBICO) was used at this stage for 7 days.

To promote the further differentiation of NPCs, on day 21, the medium was replaced with DMEM/F12 medium with 1% FBS but maintained without bFGF, RA, or Pur for 7 days.

To generate OPCs, on day 15, the NPCs were cultured in DMEM/F12 supplemented with 10 ng/mL bFGF, 10 ng/mL platelet-derived growth factor-AA (PDGF-AA; Invitrogen), 1% N2 and 2% KSR for up to 28 days. Then, PDGF-AA and bFGF were excluded from the OPC medium, and 3,3′5′-triido-L-thyronine (T3, 30 ng/ml; Sigma) and 10% FBS were added to differentiate the OPCs into oligodendrocytes.

### Preparation of miR-219 lentivirus expression vector and transfection

The miR-219 locus on chromosome 2 and its approximately 200 bp flanking sequences were PCR amplified from mouse genomic DNA and inserted into the GV254 vector (the functional element is Ubi-EGFP-MCS-IRES-Puromycin) to form miR219-GV254 recombinant plasmids. To generate the scrambled miRNA construct, the control sequence “TTCTCCGAACGTGTCACGT” was inserted. Recombinant lentivirus was prepared by transient cotransfection of HEK 293 T cells with the appropriate transfer vector and lentiviral helper plasmids (20 μg pGC-LV, 15 μg pHelper 1.0, 10 μg pHelper 2.0) using Lipofectamine 2000 transfection reagent (Invitrogen) according to the manufacturer’s instructions. After 8 h of incubation, the medium was exchanged with fresh medium. On the following day, the lentivirus was harvested, filtered through a 0.45-μm filter and concentrated by ultracentrifugation (140 min at 50,000 × *g*). Viruses were titered by flow cytometry and then used to infect HEK 293 T cells. Titers for the preparations used here were 2 × to 5 × 10^8^ infectious units/ml. mESCs were infected with LV-miR-219 at a multiplicity of infection (MOI) = 100 when mESCs grew until 80–90% confluent in serum-free Opti-MEM (GBICO).

### RNA extraction and RT-qPCR

To use qRT-PCR to analyze miR-219 expression, RNA samples were obtained from mESCs cultured for 3 days. Taqman miRNA RT-qPCR analysis was performed on 10 ng of RNA for the indicated miRNAs, following the manufacturer’s protocol (Applied Biosystems) and normalized to U6 snRNA. SYBR Green incorporation RT-qPCR was used to detect mouse mRNA expression as performed on an ABI StepOnePlus Real Time PCR system following the manufacturer’s instructions, then normalized to GAPDH mRNA levels.

To use RT-PCR to analyze gene expression, RNA was purified from mESCs, NPCs and terminally differentiated cell samples using the Qiagen RNeasy Mini Kit, and 400 μg of total RNA was reverse-transcribed using a First Strand cDNA Synthesis kit (Takara). The data were analyzed using the ddCt method. All primers are provided in Table 1.

### OPCs-spinal cord explant co-culture

The miR219-OPCs and spinal cord explant co-culture was conducted as described previously^65^. Glass coverslips treated with 25 mM HCl were coated in 2% Matrigel in 6-well plates. OPCs derived from miR219-mESCs were cultured for 8–10 days prior to co-culture following above protocols. Embryonic Sprague–Dawley (SD) rats at 14.5–16 days of gestation were removed from their pregnant mothers and transferred to a 100-mm Falcon culture dish that contained Hanks’ balanced salt solution (HBSS; Invitrogen). Their vertebral canals were opened and spinal cords were removed rapidly into another 100-mm dish that contained HBSS, using sterile fine-tipped forceps and micro-spring scissors. After the meninges and blood vessels were removed carefully, the embryonic spinal cords were cut into small pieces (no more than 0.5 mm in length) by scissors, which would be used as spinal cord explants. Spinal cord explants were plated onto miR219-OPCs and allowed to grow for 2 weeks before fixation and immunolabeling. The co-culture medium consists of DMEM/F12 and Neurobasal (1:1), 1% N2 medium, 2% B27, 2 mM glutamine (GBICO), 0.1% BSA, 10 ng/ml biotin and 30 ng/ml T3 (Sigma).

### Immunocytochemistry

mESCs and differentiated cultures were fixed with 4% paraformaldehyde (Sigma) and permeabilized with 0.3% Triton™ X-100 (Sigma) followed by 1 h of blocking with 10% goat serum (Sigma) and were incubated overnight with primary antibodies at 4 °C. The following primary antibodies were used: for ESC, Nanog (rabbit IgG, 1:500, Abcam) and Oct4 (rabbit IgG, 1:200, CST); for NPC, nestin (mouse IgG1, 1:1000, Abcam) and musashi1 (rabbit IgG1, 1:100, Sigma); for neurons, β3-tubulin (Tuj1, rabbit IgG, 1:100, Santa Cruz Biotechnology, USA); for astrocytes, glial fibrillary acidic protein (GFAP; rabbit IgG, 1:300, Abcam); for oligodendrocytes, Galc (mouse IgG1, 1:500, Abcam), cyclic nucleotide 3′ phosphodiesterase (CNPase; mouse IgG1, 1:100, Santa Cruz Biotechnology, USA) and myelin basic protein (MBP; mouse IgG1, 1:500, Covance); for OPCs, A2B5 (rabbit IgG, 1:200, Abcam) and NG2 proteoglycan (rabbit IgG, 1:200, Millipore); for pre-oligodendrocytes, O4 (mouse IgG1, 1:200, Sigma); and for neuron axons, NF-200 (rabbit IgG1, 1:500, Abcam). For negative controls, cells were incubated with PBS instead of the primary antibodies. Subsequently, incubation with secondary antibodies was performed for 2 h at room temperature. The secondary antibodies used were: goat anti-mouse IgG (H + L) Alexa Fluor ^®^555 conjugated or goat anti-rabbit IgG (H + L) Alexa Fluor ^®^555 (1:500, Invitrogen, Eugene, OR, USA). Nuclei were stained with 4,′6-diamidino-2-phenylindole, DAPI (Sigma, USA). Fluorescence images were captured using a Zeiss Axioskop 40 microscope (Carl Zeiss, Oberkochen, Germany).

### Animals and administration

Eight-week-old wild-type C57BL/6 male mice were purchased from Shanghai Laboratory Animal Center (Shanghai, China). Experimental chronic demyelination was induced by feeding mice a diet containing 0.2% (w/w) cuprizone [bis–cyclohexanone-oxaldihydrazone] (Sigma) mixed into a ground standard rodent chow for 12 weeks^41^. Untreated control mice were maintained on a diet of normal pellet chow.

For transplantation, the cuprizone-induced mice were anesthetized with 3% isoflurane in a mixture of air and 100% oxygen (1:1) and positioned in a stereotaxic apparatus. They then received a single injection of 2 μl scramble-OPCs (scramble group, n = 17) or miR219-OPCs [miR-219 group, n = 17, (1 × 10^5^ cells/μl Hank’s Balanced Salt Solution, HBSS)] injected for 0.2 μl/min at the coordinates: anterior-posterior +0.5, medial-lateral ±1.2, and dorsal-ventral −2.5 (Paxinos and Franklin, 2001). The vehicle group received an injection of 2 μl HBSS.

For the immunosuppressive therapy protocol, OPC-transplanted mice received tacrolimus (TAC; Sigma- Aldrich) and sirolimus (SIR; Rapamune oral solution; Sigma-Aldrich). TAC and SIR were administered once daily by oral gavage, 4 mg/kg/d for TAC, and 3 mg/kg/d for SIR. To label proliferating cells, bromodeoxyuridine (BrdU, 50 mg/kg; Sigma, St. Louis, MO, USA) was injected intraperitoneally daily during the last 3 days before perfusion. All procedures in the experiment were consistent with Chinese legislation on the use and care of laboratory animals and were approved by the Xuzhou Medical University committee for animal experiments.

### Behavioral tests

All of the behavioral experiments were performed during the seventh week after transplantation. Each group contained eight mice.

### Step-through passive avoidance task

The step-through passive avoidance apparatus consisted of an illuminated chamber (11.5 cm × 9.5 cm × 11 cm) lit with a 25 W lamp, attached to a darkened chamber (23.5 cm × 9.5 cm × 11 cm) containing a metal floor that was capable of delivering a mild electric shock (0.3 mA, 50 Hz, 5 s). A guillotine door separated the two compartments. The step-through test was performed as previously described^66^,^67^. Briefly, mice were placed in a dimly lit room containing the apparatus for 0.5 h before training, to allow them to acclimatize to the new environment. Each mouse was placed into the illuminated chamber, facing away from the door into the dark chamber, and allowed to acclimatize for 1 min. As soon as the mouse entered the dark chamber, the door was slid back into place, triggering an electric shock. The mouse was immediately removed from the chamber and returned to its cage. The latency (time used to change compartments) was recorded. The retention test was conducted 24 h later with the mouse again being placed in the illuminated chamber and subjected to the same protocol but without the electric shock. The upper time limit was set at 300 s.

### Morris Water Maze test

The Morris Water Maze (MWM) test was performed as previously described^66^,^67^.The experimental apparatus consisted of a circular water tank (100 cm in diameter, 35 cm in height), containing water (23 ± 1 °C) to a depth of 15.5 cm, which was rendered opaque by adding milk powder. A platform (4.5 cm in diameter, 14.5 cm in height) was submerged 1 cm below the water surface and placed at the midpoint of one quadrant. The pool was located in a test room that contained various prominent visual cues. Each mouse received four training periods per day for 4 consecutive days. The latency to escape from the water maze (by finding the submerged escape platform) was calculated for each trial. On day 5, the probe test was performed by removing the platform and allowing each mouse to swim freely for 60 s. The time that the mice spent swimming in the target quadrant (where the platform was located during hidden platform training) and in the three nontarget quadrants (right, left and opposite quadrants) was measured, respectively. For the probe trials, the number of times each mouse crossed over the platform site was also measured and calculated. All data were recorded with a computerized video system.

### Tissue preparation

Mice from each group at 1 week (n = 3), 2 weeks (n = 3), 4 weeks (n = 3) and 8 weeks (n = 4) after transplantation were intracardially perfused with PBS followed by fixation with 4% cold paraformaldehyde. The brains were removed and post-fixed in 4% paraformaldehyde for 6 h at 4 °C, incubated overnight at 4 °C in 100 mM sodium phosphate buffer (pH 7.4) containing 30% sucrose, and then embedded in Optimal Cutting Temperature medium (Leica Microsystems, Nussloch, Germany) for cryosectioning. Coronal sections (10 μm or 20 μm) from the bregma anterior-posterior +1.0 to −1.0 were cut and collected on 3-aminopropyltriethoxysilane-coated slides (Sigma-Aldrich) and stored at −80 °C. Ten-micron composite sections were stained routinely with LFB-PAS, and 20 μm sections were stained by immunohistochemistry.

### Histology and immunohistochemistry

The extent of myelination in the corpus callosum was examined by staining coronal brain sections with Luxol Fast Blue and Periodic Acid-Schiff (LFB-PSA, Sigma). The sections were scored based on the relative ratio of blue or pink fibers detected visibly by the observer, on a scale from zero to three, with three being completely myelinated and zero being completely demyelinated. Intermediate scores in 0.25 increments reflect partial demyelination^68^.

For immunohistochemistry, primary antibody NG2 proteoglycan (rabbit IgG, 1:200, Millipore), myelin associated glycoprotein (MAG, rabbit IgG, 1:100, Santa Cruz Biotechnology, USA), MBP (mouse IgG, 1:500, Covance), GFAP (rabbit IgG, 1:300, Abcam), Iba-1(rabbit IgG, 1:1000, Wako), BrdU (rabbit IgG, 1:1000; Abcam, Cambridge, UK) and nestin (mouse IgG,1:1000; BD PharMingen, Inc., San Diego, CA, USA) were used. For negative controls, sections were incubated with PBS instead of the primary antibodies. After PBS washes, sections were incubated for 1 h with secondary antibodies: goat anti-mouse IgG (H + L) Alexa Fluor ^®^555 conjugated or goat anti-rabbit IgG (H + L) Alexa Fluor ®555 (1:500, Invitrogen, Eugene, OR, USA). The specificity of the staining was assessed by omitting the primary antibody. Slides were mounted with Mowiol (Calbiochem, USA) containing DAPI (Sigma, USA) according to the manufacturer’s instructions. For BrdU staining, sections were pretreated with 50% formamide/280 mM NaCl/30 mM sodium citrate at 65 °C for 2 h, incubated in 2 M HCl at 37 °C for 30 min, and rinsed in 0.1 M boric acid (pH 8.5) at room temperature for 10 min.

The counting of NG2/GFP or MAG/GFP double-positive cells in the corpus callosum was performed in three sections per animal from the same levels, at every 9–12^th^ section between bregma levels +1.0 and 0.02 mm. Areas of interest were scanned with a 20 × objective lens in 600 μm × 450 μm format in the *x*–*y* direction. The number of NG2/GFP or MAG/GFP double-positive cells in each field was then converted into the number of double-positive cells/mm^2^. To determine the number of BrdU- or nestin-labeled cells in the anterior subventricular zone (SVZa), we selected every 13–15^th^ section between bregma levels +1.0 and 0.02 mm (total = 3 sections per brain). In each section, a rectangle of 200 μm × 100 μm per side was placed within the SVZa, and the total number of positive cells per rectangle was counted^69^.

For quantitative analysis of the MBP immunofluorescence staining, integral optical density (IOD) and a region of interest were measured by Image-Pro Plus 6.0 software (Media Cybernetics Inc., USA). Values (three slides for each brain) of optical density in individual cells represented the quantity of objective protein and were calculated using the following equation: ΣIOD/ΣArea, where IOD is the integral optical density in a region of interest, and area is the area of a region of interest. In this study, ΣIOD is the sum of the IOD of MBP/GFP double-positive cells in the photograph, and ΣArea is the total area of MBP-positive cells in the photograph^64^.

### Electron microscopy

For electron microscopic examination, Epon embedding was performed as previously described^70^. In brief, four animals per group were transcardially perfused with 2% glutaraldehyde (Gla) and 2.5% PFA in 0.1 M PBS. Brains were quickly removed and placed on ice. The corpus callosum was dissected, and the samples were placed in a tube. Immediately after extraction, samples were placed in 3% Gla in 0.1 M cacodylate buffer (pH 7.4) at 4 °C overnight and transferred to 1% osmium tetroxide in the same buffer for 1 h at room temperature. Tissue was transversely cut into 1 mm blocks that were fixed in osmium tetroxide at 4 °C overnight, dehydrated through ascending ethanol washes, and embedded in epoxy resin. To study the remyelinated axons of the corpus callosum, serial 1 μm-thick semi-thin sections were cut with a diamond knife, stained with 1% toluidine blue, and examined by light microscopy. To analyze myelin sheaths in the corpus callosum, 60–70 nm-thick ultra-thin sections were stained with uranyl acetate and lead citrate prior to examination by tEM (FEI Tecnai G2 T12, USA), and the image was analyzed by TEM Imaging and Analysis, TIA.

For morphometric analysis, we measured at least 100 axons. We counted the total number of axons with and without myelin sheaths contained within all of the selected electron micrographs from each animal. We measured the axonal diameter (d) as the shortest distance across the center of the axon, avoiding the myelin sheath thickness. The axonal diameter plus the total myelin sheath thickness on both sides was defined as the fiber diameter (D). The *g*-ratio was calculated using the d/D ratio. Therefore, a completely demyelinated fiber would have a *g*-ratio = 1, and myelinated fibers would have a ratio of <1. The *g-*ratio was calculated for each myelinated fiber, then the average of all the g-ratio values from one brain was calculated. The mean of the average *g*-ratios from the three brain samples was determined for each group^71^,^72^.

### Statistical analysis

All statistical analyses were performed with SPSS software, version 11.5. Group differences in the escape latency in the MWM training task were analyzed using two-way ANOVA. The other data were analyzed using Student’s *t*-test or one-way ANOVA. The data are expressed as the mean ± s.e.m. Statistical significance was set at *P* < *0.05* for all tests.

## Additional Information

**How to cite this article:** Fan, H.-B. *et al*. Transplanted miR-219-overexpressing oligodendrocyte precursor cells promoted remyelination and improved functional recovery in a chronic demyelinated model. *Sci. Rep.* **7**, 41407; doi: 10.1038/srep41407 (2017).

**Publisher's note:** Springer Nature remains neutral with regard to jurisdictional claims in published maps and institutional affiliations.

## Figures and Tables

**Table 1:** Primer sequences for RT-PCR analysis.

Gene (mouse)	Primer sequence (5 → 3)	Length (bp)	Accession No.	AT (° C)			
Nanog	F 5’-AACGCCTCATCAATGCCTGC-3’R 5’-CCTTGTCAGCCTCAGGACTTG-3’	186	AB093574.1	60			
Oct4	F 5’-GGAGCTAGAACAGTTTGCCAAG- 3’R 5’ -GAAGCGACAGATGGTGGTCT-3’	130	BC068268.1	59			
nestin	F 5’- TCAACCCTCACCACTCTATTTT-3’R 5’-GCTGTTTTCTACTTTTACCTCTGTG -3’	143	NM_016701.3	57			
Musashi1	F 5’-AAGGTGGATGATGCCATGCT-3’R 5’-CCAGCATGAAGGCATCCATT-3’	230	NM_008629.1	58			
GFAP	F 5’-GTCTCGAATGACTCCTCCACT-3’R 5’-CGGACCTTCTCGATGTAGCT-3’	146	NM_010277.3	58			
Tuj1	F 5’-ACGCATCTCGGAGCAGTT-3’R 5’-CGGACACCAGGTCATTCA -3’	125	NM_023279.2	56			
Galc	F 5’-TTATTGTTGTGTGCGCTGCT-3’R 5’ -TTACTAGAAGCCGGGAGGTTG-3’	126	NM_008079.4	58			
