# Author Correction: Transplanted miR-219-overexpressing oligodendrocyte precursor cells promoted remyelination and improved functional recovery in a chronic demyelinated model

**DOI:** 10.1038/s41598-018-34063-w

**Published:** 2018-10-29

**Authors:** Hong-Bin Fan, Li-Xia Chen, Xue-Bin Qu, Chuan-Lu Ren, Xiu-Xiang Wu, Fu-Xing Dong, Bao-Le Zhang, Dian-Shuai Gao, Rui-Qin Yao

**Affiliations:** 1 Department of Cell Biology and Neurobiology, Xuzhou Key Laboratory of Neurobiology, Jiangsu Key Laboratory of New Drug Research and Clinical Pharmacy, Xuzhou Medical University, Xuzhou, 221009 China; 2 Department of Neurology, Affiliated Hospital of Xuzhou Medical University, Xuzhou, 221002 China; 3 Clinical Laboratory, Xuzhou TCM Hospital Affiliated to Nanjing University of Chinese Medicine, Xuzhou, 221000 China; 4 Department of Laboratory, No. 100 Hospital of CPLA, Suzhou, 215007 China

Correction to: *Scientific Reports* 10.1038/srep41407, published online 01 February 2017

This Article contains an error in the affiliation for Li-Xia Chen, which is incorrectly listed as ‘Clinical Laboratory, Xuzhou Center Hospital, Xuzhou, 221000, China’. The correct affiliation is listed below:

Clinical Laboratory, Xuzhou TCM Hospital Affiliated to Nanjing University of Chinese Medicine, Xuzhou, 221000, China.

